# Morphological variations of the dural venous sinuses: a cadaveric study

**DOI:** 10.3389/fsurg.2026.1800026

**Published:** 2026-05-15

**Authors:** Burcu Kamaşak Arpaçay, Kenan Aycan

**Affiliations:** Department of Anatomy, Faculty of Medicine, Kırşehir Ahi Evran University, Kırşehir, Türkiye

**Keywords:** brain, dural venous sinuses, human cadaver, hypoplasia, variation

## Abstract

**Introduction:**

The dural venous sinuses are essential for cerebral venous drainage and exhibit notable anatomical variability, particularly at the confluence of sinuses (CS). Such variations may mimic pathological conditions, affect radiological interpretation, and influence surgical planning. This study aimed to evaluate anatomical variations of the dural venous sinuses, with emphasis on features relevant to the diagnosis of venous pathologies.

**Methods:**

An anatomical study was conducted on 15 adult cadaver brains (five female, 10 male; mean age 70 years). Following routine scalp and calvarial dissection, dural venous sinuses in the region of the CS were carefully exposed and preserved. The presence, drainage patterns, relative positions, and widths of the superior sagittal sinus (SSS), transverse sinuses (TS), straight sinus (StS), occipital sinus (OS), and CS were documented. Measurements were obtained using a digital caliper and averaged from repeated assessments.

**Results:**

Marked variability was observed in the anatomy of the dural venous sinuses. The CS was absent in 20% of cases. Asymmetry, hypoplasia, or absence of individual sinuses—including the OS, TS, or StS—was frequently encountered. Multiple drainage patterns of the SSS and StS, dural folds replacing the CS, and accessory sinuses were identified. Mean sinus widths were 6.73 ± 2.34 mm for the SSS, 5.71 ± 2.45 mm for the left TS, and 5.33 ± 2.05 mm for the right TS.

**Conclusion:**

Awareness of dural venous sinus variations is crucial to avoid misdiagnosis of venous pathology and optimize radiological assessment and surgical planning in posterior cranial fossa and cerebrovascular procedures.

## Introduction

The dural venous sinuses constitute a vital component of the cerebral venous drainage system, intricately woven within the layers of the meninges to facilitate the return of deoxygenated blood from the brain to the systemic circulation. Venous blood from the head and neck region, together with cerebrospinal fluid, drains into the dural venous sinuses. The human intracranial venous system has unique anatomical characteristics and varies among individuals. The dural venous sinuses—positioned between the two layers of the dura mater—show less variability than the cerebral cortical venous system (1).

The confluence of sinuses (CS)—also called the Torcular Herophili—is the connecting point of the superior sagittal sinus (SSS), straight sinus (StS), and occipital sinus (OS) and receives venous drainage from various regional dural venous sinuses (2). The CS is defined by the union of the SSS, StS, OS, and the bilateral transverse sinus (TS). In cases of rightward deviation, the right TS originates from the CS. Moreover, the CS accepts venous drainage from the OS and maintains midline communication with the left TS (3).

Irregular growth patterns can lead to varying height and size asymmetry of the dural sinuses as well as mild to marked irregularities (2, 3). Some variations in the dural venous sinuses mimic sinus vein thrombosis and mass invasions, leading to misdiagnosis. Knowledge of these anatomic variations may prove useful during preoperative evaluation and radiological reporting (4).

Many anatomic and cadaveric studies have investigated dural venous sinuses and CS (2, 5, 6). These studies have been carried out to map the venous drainage pattern around the CS and to understand the variations of the dural venous sinuses in the posterior cranial fossa approach during the surgical treatment of cerebrovascular diseases and brain tumors (4, 7–11). Congenital asymmetry and hypoplasia of dural sinuses are important in terms of dural sinus thrombosis (7).

Understanding variations of the dural venous sinuses is important for surgeons during interventions as they help distinguish between pathological structures and normal anatomy. In particular, the anatomical variants of dural venous sinuses can influence surgical planning and may be one of the factors to consider during surgical intervention (9). The aim of this study was to evaluate the variations of dural venous sinuses that should be considered in the diagnosis of venous pathologies.

## Materials and methods

This study was based on naked-eye examination of the dural venous sinuses in the region of the confluence of sinuses in 15 adult cadavers (five female, 10 male). The cadavers ranged in age from 60 to 76 years, with a mean age of 70 years. We used photographs of 15 adult cadaver brains exhibiting anatomical variations encountered during routine brain dissection. This study was conducted in accordance with institutional and international ethical standards for the use of human cadaveric materials. It was reviewed and exempted from ethics committee approval by Kırşehir Ahi Evran University Health Sciences Research Ethics Committee (Number: 715175, Date: 7 January 2025). All experiments were performed in accordance with the Declaration of Helsinki. All cadaveric specimens were obtained through body donation programs and were used solely for educational and scientific purposes, with no identifiable personal data included. Since no data allowing the identification of individuals were presented, there were no ethical concerns in this study. All cadavers were fixed with 10% formaldehyde solution. The scalps and calvarias of the cadavers were dissected using routine dissection methods. None of the cadavers died from head injury, and no anomalies or damage was observed in the vascular system or head and neck. Bicoronal scalp dissection was initiated from the parietal bones and extended to the posteroinferior aspect of the skull. The calvaria were subsequently removed using a high-frequency whorl device. Special attention was paid to the preservation of the dural sinuses during the dissection of the dura mater. The dura mater was carefully microdissected from the bone tissue. The dural venous sinuses of the posterior fossa were observed and photographed. Then, the location and width of each dural venous sinus were noted. Measurements of the dural venous sinuses were obtained using a digital caliper, performed by the same person at least two times, and the average was noted. In this study, the following anatomic measurements were completed:

1. The width of all existing dural venous sinuses was measured.
2. The drain location of the SSS was identified.
3. The superior–inferior location of bilateral TSs was determined.
4. The internal structures of the CS and the drainage openings of the straight sinus (StS) were examined.
5. The presence and size of the OS were evaluated.
6. Other variations were identified.

### Statistical analysis

Data normality was assessed using histogram and Q-Q plots, along with the Shapiro–Wilk's test. Descriptive statistics for the variables were summarized as mean ± standard deviation. Confidence intervals (CI) were calculated to estimate the precision of the results. A 95% confidence level was used, and the intervals were computed based on the standard error of the mean. The relationships between variables were determined using the Pearson correlation coefficient, based on the normality assumption. All statistical analyses were performed using the Statistical Package for Social Sciences, version 29.0 for Windows software (IBM SPSS Statistics for Windows, Version 29.0. IBM Corp., Armonk, NY, USA).

## Results

**Figure 1:**
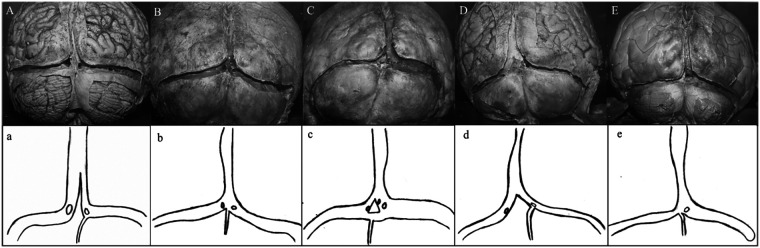
Diagrams of brains **(A–E)** of dural venous sinus configurations, highlighting variations in CS morphology, SSS branching, and TS asymmetry.

**Figure 2:**
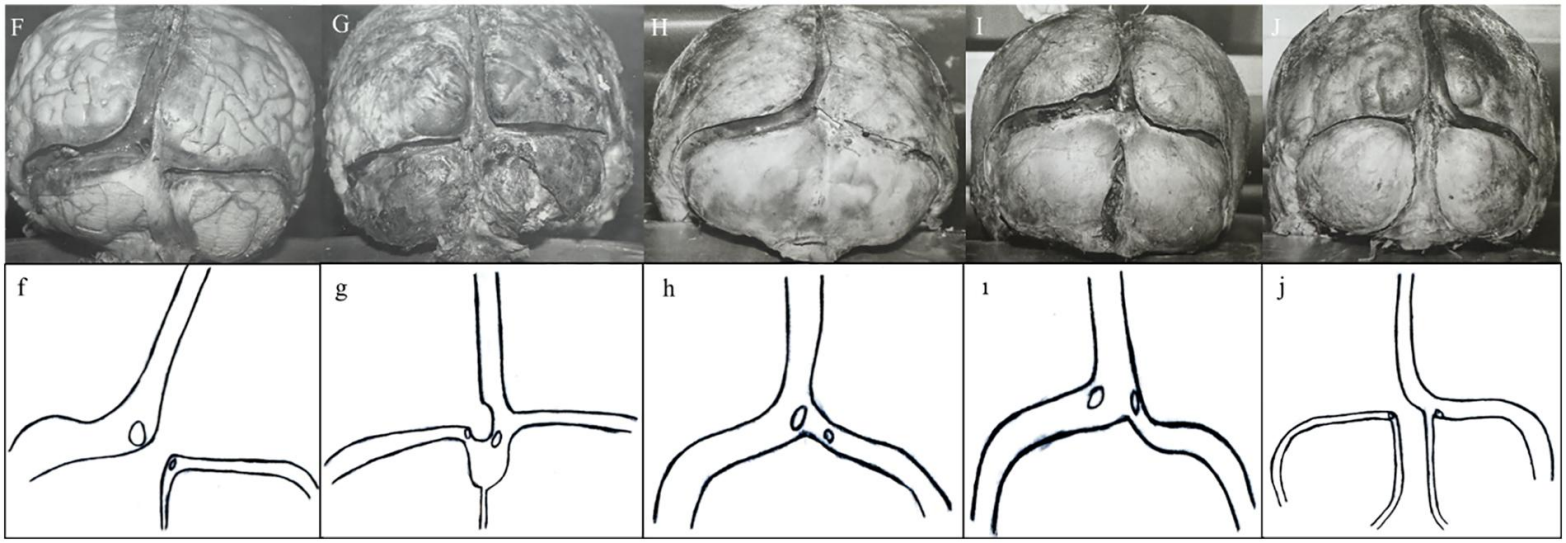
Diagrams of brains **(F–J)** demonstrating absence of CS, alternative drainage pathways, and variations in StS and OS configurations.

**Figure 3:**
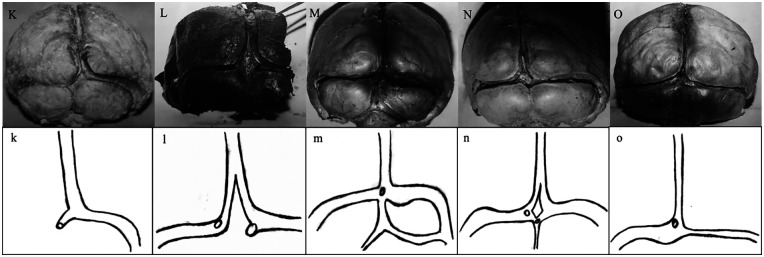
Diagrams of brains **(K–O)** emphasizing rare configurations, including accessory sinuses, dural fold replacement of CS, and asymmetrical venous patterns.

As a result of anatomical dissection with preservation of sinuses in the supratentorial and infratentorial regions, the SSS, CS, TS, straight sinus (StS), and some OS were observed. The CS was absent in three (20%) of 15 cases. In seven cases (46.66%), the CS consisted of four sinuses: SSS, left TS, right TS, and OS. In four cases (26.66%), the OS was absent. In one case (6.66%), the left TS was absent. In one case (6.66%), the StS was absent. The results of the 15 cases are summarized as follows (Figures 1–3):

*Brain A:* The SSS (8 mm) was divided into two branches when it reached the CS. The right branch joined the right TS, and the left branch joined the left TS, continuing on both sides as the TS. The widths of the right and left TS were the same (6 mm) and both were almost aligned. In place of the CS, a piece of dura mater (triangular in shape and 3.5 cm in height) was observed. The StS divided into two terminal branches, opening into the right and left TSs. The OS (1.5 mm) drained into the right TS (Figure 1).

*Brain B:* The SSS (10 mm) joined the CS normally. The right TS (7 mm) was wider than the left TS (6 mm). The left TS was located higher than the right TS. The StS drained into the CS through two openings. The OS (1 mm) drained into the CS (Figure 1).

*Brain C*: The SSS (7 mm) divided into two branches as it entered the CS; the right branch opened into the right TS, while the left branch opened into the left TS. The right TS was located higher than the left TS. In the middle of the CS, a triangular piece of dura mater was observed. The shortest distances from this dural structure to the left TS, right TS, and OS were 2 mm, 4 mm and 8 mm, respectively. The right TS (10 mm) was wider than the left TS (4 mm). The StS opened into the CS through three openings. The OS (1 mm) drained into the CS (Figure 1).

*Brain D:* The SSS (6 mm) divided into right (3 mm) and left (2 mm) branches as it drained into the CS; these branches joined the right and left TS (each 5 mm), respectively. The widest part of the SSS before it split into two branches measured 15 mm. The right TS was located higher than the left TS. The StS drained into the TSs through two openings. The OS (1 mm) drained into the right TS. In place of the CS, a piece of dura mater was observed (Figure 1).

*Brain E:* The SSS began wide (10 mm) and then narrowed (6 mm) as it approached the CS. The SSS drained into the CS normally. The right TS (6 mm) was wider than the left TS (4 mm). The right TS was located higher than the left TS. The OS drained into the left TS. The StS drained into the CS through a single opening (Figure 1).

*Brain F:* The CS was absent. The SSS (8 mm) continued as the left TS. The left TS (10 mm) was significantly wider than the right TS (3 mm). The left TS was located higher than the right TS. The OS (1.5 mm) joined the right TS and then continued as the right TS (3 mm). As the StS approached the site of the CS, it divided into two branches, opening into the right and left TS (Figure 2).

*Brain G:* The SSS (6 mm) drained into the CS normally, but a dura mater fragment was present on the left side of the entry point. The SSS continued as the right TS. The CS widened toward the OS, which joined at this site. The OS (0.5 mm) drained into the CS. The TSs were located on the right and left sides of this widened area. The right TS (5 mm) was wider than the left TS (4 mm). The right TS was located higher than the left TS. The StS drained into the CS via two distinct openings (Figure 2).

*Brain H:* After the SSS (5 mm) merged with the CS, it continued as the left TS (8 mm). The right TS (2 mm) continued as a very thin extension. The left TS was located higher than the right TS. The StS divided into two openings, with the left one draining into the CS and the right one draining into the right TS. The OS was not evident (Figure 2).

*Brain I:* The SSS (8 mm) drained into the CS normally. The right and left TSs arose from the CS and continued onward. The left TS (10 mm) was wider than the right TS (4 mm). The left TS was located higher than the right TS. As the right TS approached the sigmoid sinus, its diameter increased (at first: 2 mm, at last: 4 mm). The OS was significantly wider (7 mm) and opened deeply into the CS. The StS divided into two branches, opening into the right and left TSs (Figure 2).

*Brain J:* The CS was absent. The SSS (6 mm) joined the right OS and continued as the right TS (4 mm). The left TS (1.5 mm) had no connection with the right TS. Two OSs were identified, with the right OS (1.5 mm) draining into the right TS and the left OS (1 mm) draining into the left TS. The right TS was located higher than the left TS. The StS drained separately into each TS through two openings (Figure 2).

*Brain K:* The SSS (8 mm) continued as the right TS (5 mm). The CS, left TS, OS, and StS were absent. Since there was no left TS, alignment comparison with the right TS could not be performed (Figure 3).

*Brain L:* As the SSS (9 mm) approached the CS, it bifurcated into right (4 mm) and left (1.5 mm) branches, which continued as the right (6 mm) and left (4 mm) TSs, respectively. The right TS was located higher than the left TS. A triangular piece of dura mater was present at the location of the CS. The StS drained separately into both TSs through two distinct openings. The OS was not clearly identifiable (Figure 3).

*Brain M:* The SSS (5 mm) drained into the CS in a typical manner. The right TS began wide (11 mm) and then narrowed (8 mm), remaining wider than the left TS (4 mm). The right and left TSs were nearly aligned. Two accessory sinuses were present in this brain, parallel to the TS and originating from the OS. The OS expanded (10 mm) and connected the accessory sinus with the CS. The right accessory sinus (4 mm) started from the OS and ended at the junction of the TS and the sigmoid sinus. The left accessory sinus (2 mm) ended blindly. The StS drained into the CS through a single opening (Figure 3).

*Brain N:* As the SSS (3 mm) approached the CS, it divided into two branches, draining into the right and left TSs. The right TS narrowed (4 mm) and then widened (6 mm). The left TS started narrow (3.5 mm) and then widened (7.5 mm). The right and left TSs were nearly aligned. At the expected location of the CS, a quadrilateral dural fold was present (length: 2.5 cm; width: 1 cm). The StS opened through two separate apertures, one draining into the left TS and the other into the junction of the OS (1.5 mm) with the CS. The OS drained into the CS in a typical manner (Figure 3).

*Brain O:* The SSS (2 mm) drained into the CS in a typical manner. The right and left TSs continued directly from the CS, with the left TS (6 mm) being wider than the right TS (3 mm). The left TS was located higher than the right TS. The StS drained into the CS through a single aperture. The OS was absent (Figure 3).

The most frequent findings included asymmetry of TSs, frequent bifurcation of the SSS, and variability in StS drainage. Absence or replacement of the CS was observed in a notable proportion of specimens, and accessory sinuses were identified in rare cases. These findings highlight the heterogeneity of venous sinus anatomy.

The mean widths of the measured dural venous sinuses were as follows (mm): SSS (6.73 ± 2.34; 95% CI: 5.20–8.03), left TS (5.71 ± 2.45; 95% CI: 4.29–7.13), and right TS (5.33 ± 2.05; 95% CI: 4.12–6.58). No significant relationship was found between the SSS and the left TS (*r* = −0.025, *p* = 0.932) or the right TS (*r* = 0.257, *p* = 0.356). Similarly, no significant relationship was found between the left TS and the right TS (*r* = −0.449, *p* = 0.107). As other sinuses were not present in all brains, mean measurements could not be calculated for them.

### Proposed simplified classification of CS patterns

Based on our findings, we propose a simplified classification of CS patterns with surgical relevance:

Type I: Complete confluence: This is a classical configuration in which all major sinuses are joined.

Type II: Partial confluence: This signifies an incomplete communication between sinuses.

Type III: Absent confluence: The sinuses drain independently or via alternative channels.

Type IV: Dural fold replacement: The CS is replaced by a fibrous dural partition.

This classification may provide a practical framework for surgeons in preoperative planning and risk assessment.

## Discussion

Given the complex architecture of the dural venous system, it is susceptible to a wide range of pathological and anatomical variations. For accurate differentiation of these pathological conditions, the variations must be well understood. The dural venous sinuses enable drainage of CSF through arachnoid villi. Damage to the dural venous sinuses can impede CSF transport and lead to intracranial hypertension. The dural venous sinuses also drain blood from the cerebral cortex and white matter via the superficial cortical veins. Pathologies affecting cortical veins also cause an increase in venous capillary pressure, disruption of the blood–brain barrier, and impaired drainage of blood from the brain tissue. Therefore, conditions such as occlusion can result in brain tissue damage (9, 12).

The findings of our study support the heterogeneity reported in the literature, particularly regarding the asymmetry of the TSs and variations in the drainage patterns of the SSS and StS. Moreover, in one case, the CS, left TS, OS, and StS were all absent. Our findings strongly support previous reports (2, 13), indicating that the classical textbook representation of a symmetrical CS is uncommon in humans. Instead, a spectrum of drainage patterns, asymmetries, and even complete absence of the CS were noted, consistent with the descriptions of Browder and Kaplan (14). In addition to cadaveric studies on dural venous sinus variations, studies using MRI, CT, and other techniques are available in the literature. However, the novelty of the present study lies in its comprehensive documentation of combined anatomical patterns observed within individual specimens. These findings reinforce and extend existing anatomical and radiological literature by demonstrating the complexity and coexistence of multiple variations within the same specimen.

### Variations in the superior sagittal sinus

Bisaria (2) documented several examples of SSS division into right and left components before entering the TSs, attributing this configuration to the persistence of embryonic plexiform venous networks. An important observation in the our study was the frequent bifurcation of the SSS as it approached the CS, as seen in brains A, C, D, L, and N. The prevalence of such bifurcation patterns appears to be higher in direct anatomic studies than in radiological series (15). Our findings therefore suggest that bifurcation of the SSS may be more common than previously recognized.

In the dural venous sinus study conducted by Kopuz et al. (16) on 33 newborn cadavers, the terminal shapes of SSS were classified into six types: Type I: 30.3%, Type II: 21.2%, Type III: 12.1%, Type IV: 6.1%, Type V: 9.1%, and Type VI: 21.2%. Applying this classification to our study, the distribution was as follows: Type I: 0%, Type II: 6.66%, Type III: 13.33%, Type IV: 0%, Type V: 33.33%, and Type VI: 20%. Three cases could not be included in this classification due to the absence of OS, and three cases had previously unseen variations.

San Millán Ruíz et al. (17) reported that three of seven patients with a unilateral hypoplastic rostral SSS also had at least another dural venous sinus anomaly. Nashed et al. (18) reported that 89.9% of 169 patients had Type I SSS [According to San Millán Ruíz et al. (17)], and approximately 10% of patients had abnormal configurations.

In our study, in several specimens (A, C, D, L, and N), the bifurcated SSS drained symmetrically into both TSs, while in other specimens (F, G, H, J, and K), unilateral drainage occurred. These drainage patterns align with MR venography studies, indicating that while right-sided dominance remains most common, bilateral and left-dominant drainage are not rare (7, 9). Park et al. (19) observed that in 51.6% of cadavers, the SSS drained into the right TS, and in 14 cases (45.2%), it entered via the center of the CS. Although studies (8, 15, 20, 21) have mostly shown the SSS to continue with the right TS, as seen in our study, the location where the SSS opens or continues may vary. In our study, the SSS opened into the right TS at a rate of 13.33%, to the left TS at a rate of 13.33%, and to the center of the CS at a rate of 40%. This diversity highlights the importance of careful radiological interpretation when evaluating venous occlusion or variations during surgical planning.

In a cadaver study conducted by Brockmann et al. (22) on nine cadavers and 30 patients, the maximum horizontal diameter at the coronary suture was 6.7 ± 2.0 mm in CT and 5.2 ± 1.1 mm in the cadavers. Suganya et al. (23) reported an SSS diameter in the lambda region of 5.7 ± 1.04 mm, and Oberman et al. (24) reported 8.5 ± 2.1 mm. In Larson et al.’s (25) study examining changes in dural venous sinus diameters from birth to 20 years of age, the average SSS diameter was 7.5 mm. In our study, the SSS diameter varied widely, but the average was 6.73 ± 2.34 mm, which was wider than the results reported by Brockmann et al. (22) and Suganya et al. (23), but narrower than the values of Oberman et al. (24) and Larson et al. (25). Although the values reported by Brockmann et al. (22) and Suganya et al. (23) are lower than those of our study, they are within the CI range of our study findings. Dilated SSS is most often not a pathological finding on its own; however, SSS enlargement may occur in conditions such as TS hypoplasia, jugular vein stenosis, and reconstruction after sinus thrombosis. It may also be associated with increased intracranial pressure and drainage load, dural AV fistula, and venous outflow obstruction (26). Knowledge of SSS diameters is important for neurosurgeons when planning craniotomies and burr opening placement in interhemispheric approaches.

### Variations in the transverse sinus

Studies using angiography and MR venography have confirmed that right-sided dominance of the TS is the most common pattern, whereas symmetrical TSs occur in only 20%–30% of individuals (7, 9). Consistent with the literature, the most frequent pattern in our study was right TS dominance, seen in brains B, C, E, G, I, J, L, and M. Several authors report right-sided dominance in 60%–80% of individuals (6–10, 19). However, we also identified multiple specimens with left TS dominance (F, H, I, N, and O), confirming that left-dominant or asymmetrical patterns are not uncommon. The literature shows heterogeneity in TS dominance. While some studies have found right TS dominance to be more frequent, others indicate that codominance is more common (Table 1). In our study, the number of cases with right TS dominance was higher, at 53.33%. The clinical relevance of this asymmetry is important because non-dominant TS may be hypoplastic or even aplastic—a normal variant that should not be misinterpreted as thrombosis on imaging (21).

**Table 1:** Comparison of TS dominance–codominance with other studies in the literature.

Studies	*n*	Right TS dominant (%)	Left TS dominant (%)	Codominant (%)
Singh et al. (6)	160	41	10	49
Alper et al. (7)	105	59	10	31
Durgun et al. (9)	189	43.4	19	37.6
Kopuz et al. (16)	33	51.5	18,2	30.3
Park et al. (19)	31	35.5	3.2	61.2
Widjaja and Griffin (21)	50	54	36	8
Present study	15	53.33	33.33	13.33

Kobayashi et al. (27), in a study of 549 patients, classified communications between the right and left TSs into four types: wide communication [Type A (88.1%)], narrow communication [Type B (10.2%], no communication [Type C (0.4%)], and single TS [Type D (1.3%)]. In Ishizaka's (5) study, Type B was seen in 13.5%, while Types C and D were seen in 23.1% combined. In our study, the distribution of TS types was as follows: Type A (33.3%), Type B (20%), Type C (40%), and Type D (6.66%).

In a study by Widjaja and Griffin (21), the diameters of the TS and the SSS were measured and compared. The TS was considered hypoplastic when its diameter was less than half the diameter of the SSS. These ratios were also accepted for TS hypoplasia in our study. In Widjaja and Griffin (21),'s study of 50 patients, four patients had codominant TSs, one patient had absence of both TSs, and two had hypoplastic left TSs. In our study, hypoplastic left TS was observed in brains E, J, and L (20%), while hypoplastic right TS was seen in brains F and H (13.33%). Notably, aplasia of TS was observed in Brain K (left TS absent) (6.66%). The rates of hypoplasia and aplasia in TS reported in the literature are summarized in Table 2. The variability identified across specimens reinforces the diagnostic importance of distinguishing congenital hypoplasia from pathological occlusion. It has been accepted that one reason for the frequent reporting of hypoplasia or aplasia of the TS is the increase in capacity and expansion of the right jugular system as a result of right atrial contraction (8).

**Table 2:** Comparison of hypoplasia and aplasia in TS with other studies in the literature.

Studies	*n*	Hypoplastic right TS (%)	Hypoplastic left TS (%)	Aplastic TS
Gökçe et al. (10)	394	17.53	49.09	17.05
Alper et al. (7)	105	6	39	20
Goyal et al. (11)	1,654	5.5	21.3	4.1
Nashed et al. (18)	35	7.1	20.7	-
Widjaja and Griffin (21)	50	54	4	2
Tantawy et al. (31)	363	-	22	3.6
Özkoç et al. (29)	31	9.3	27.5	38.8
Present study	15	13.33	20	6.66

Larson et al. (25) calculated the average diameter of the right TS as 6.38 mm and the left TS as 5.58 mm. Although their findings showed a lower left TS diameter and a higher right TS diameter compared with our study, their results are within the CI range of our findings.

The number of TSs and the extent of their intercommunication at the level of the CS may influence both the risk and severity of cerebral edema, particularly in the presence of intraoperative obstruction of the transverse or sigmoid sinus (28). Tardieu et al. (28) reported a case with unilateral advanced left TS along with the absence of right TS. In contrast, our study identified aplastic left TS, which may further increase the risk of venous sinus congestion, intracranial pressure, and cerebral edema.

Variations in growth patterns may result in differing heights and asymmetric dimensions of the dural sinuses, ranging from mild to pronounced irregularities and even the absence of the medial portion of the TS (21). In our study, vertical alignment differences between the TSs—right TS higher than left TS (C, D, E, G, J, and L) or vice versa (B, F, H, I, and O)—were also common. This demonstrated that the perfect alignment of TSs differs from what has been traditionally depicted. Vertical asymmetry may influence the configuration of the tentorial attachment and may also affect venous flow distribution.

### Variations in the confluence of sinuses

The variability of the CS makes its classification challenging. There are different classification schemes for CS in the literature (2, 6, 19). In the study conducted by Gökçe et al. (10), CS type was classified into three main groups: complete intersection (Type I), partial intersection (Type II, with subgroups), and non-intersecting (Type III). According to this classification, Type II was the most frequently observed type in our study, consistent with other studies (8, 10). While Type IIB and Type III findings—which are associated with higher complications risks—are generally reported at low rates, they were observed at higher rates in the study by Bayaroğulları et al. (8). In Type III termination, the absence of inter-sinus connections may lead to drainage problems following sinus injury or obstruction during craniotomy and neck dissection surgeries. In addition, as in Type IIB, where one transverse sinus is aplastic, particular attention should be paid to the existing transverse sinus during surgery.

Browder and Kaplan (14) noted that the term “confluence” is often a misnomer, as in most individuals the sinuses do not meet in a single dilated space but rather in a dural partition or series of channels. Our findings support this interpretation and provide direct anatomical evidence of CS variability. In our study, several brains exhibited absence (F, J, and K) or replacement of the CS by triangular or quadrilateral dural folds (A, C, D, L, and N).

### Variations in the straight sinus

The drainage patterns of the StS vary across different studies. Bayaroğulları et al. (8) determined that the StS predominantly drains into the left TS. Park et al. (19) reported that the StS drained into the center of the CS in 58.1% of patients and into the left TS in 35.5%. Özkoç et al. (29), in a study on 814 patients, observed StS continuation in 13 (1.6%) patients with the right TS and in 29 (3.6%) patients with the left TS. Cheng et al. (15) reported that the StS drained into the right TS, whereas the SSS bifurcated into both transverse sinuses in 13 patients (2.6%). In the same study, the StS drained into the left TS, and the SSS bifurcated into both the left and right TSs in seven patients.

Another variation related to the StS concerns its morphological form. Bayaroğulları et al. (8) conducted the StS was solitary in 268 (68%) cases, split in 95 (24.1%) cases, and fenestrated in 31 (7.9%) cases. Gökçe et al. (10)—examining 394 individuals—identified solitary, split, and fenestrated StS in 68%, 24%, and 8% of cases, respectively. In their study on 131 adult cadavers, Browder and Kaplan (14) observed a solitary StS in 85% and a split StS in 15%. In other studies (15, 20, 27), StS was observed in 23%–56%of patients.

The StS demonstrated diverse drainage configurations in our study. Bifurcation into right and left branches occurred in multiple specimens (A, F, H, I, L, and N), whereas numerous openings into the CS were found in the others (B, C, E, G, M, and O). These variations have important implications in neurosurgical access to the pineal region, where inadvertent injury to variant StS channels can result in significant morbidity.

### Variations in the occipital sinus

The incidence of OS varies across different studies. Gökçe et al. (10) reported OS in 15% of cases, while Cheng et al.’s (15) study reported it in 6%. Bayaroğulları et al. (8) detected double OS in 2 (1%) out of 211 patients. In a study by Kopuz et al. (16) involving 33 cadaveric cases, the OS was detected in all cases, with more than two OS channels draining into the CS. In Widjaja and Griffin’s (21) study, the OS was detected in nine out of 50 patients, with bilateral OS in five patients and unilateral OS in four patients. In a study conducted by Özkoç et al. (29) on 814 patients, they reported the right OS in 20 patients (2.5%), the left OS in two patients (0.2%), and duplication or triplication of the OS in five patients (0.6%). In our study, 10 out of 15 cadaver brains had a single OS (66.66%), and one had a double OS (6.66%).

Bayaroğulları et al. (8) detected OS in 59 (15%) of 211 patients and the single and midline branch was determined to be the most common. The OS was present as solitary and midline in 21 patients, as solitary and parasagittal in 26 patients (20 on the right and six on the left), as bilateral in 10 patients, as bilateral and merging in midline in one patient, and as multiple in one patient in Bayaroğulları et al.’s (8) study. The OS was seen in nine of 13 patients (69%) with left TS agenesis and in two of six patients (33.3%) with right TS agenesis. However, the OS was also absent in the brain with left TS agenesis in our study. In Zhou et al.’s (30) study on 145 individuals, it was found that 100 had normal OS and 45 had hyperplastic OS. Three cases of hyperplastic OS were encountered in our study.

The OS, traditionally considered a vestigial channel in adults, was present in most specimens and displayed considerable variation in diameter and drainage site in our study. The OS drained into the right TS (A, D, F, and J), left TS (E and J), or CS (B, C, G, I, M, and N). Notably, Brain M exhibited a markedly enlarged OS (10 mm), and Brain I showed an OS of 7 mm. An enlarged OS may function as a compensatory route in the presence of venous obstruction.

### Presence of accessory sinuses

An important and novel observation was the identification of accessory sinuses in Brain M. One of these originated from the OS and ended at the junction of the TS and the sigmoid sinus, while the other was a smaller accessory sinus that ended blindly. These previously undescribed venous channels highlight the complexity of venous drainage at the skull base. Accessory channels, though rarely documented, have been reported in isolated case studies and may represent developmental remnants of primitive venous plexuses. These structures may also serve as collateral conduits in pathological states.

### Surgical and radiological implications

The observed anatomical variations have direct implications for neurosurgical and radiological practice. The absence or replacement of the CS by dural folds may increase the risk of inadvertent sinus injury during posterior fossa or torcular exposure. Variations in transverse sinus dominance are particularly important when considering sinus sacrifice or ligation, as injury to a dominant sinus may result in catastrophic venous congestion. StS variations—including bifurcation or multiple drainage channels—are especially relevant in pineal region and tentorial surgeries, where precise anatomical knowledge is critical to avoid venous infarction. From a radiological perspective, hypoplastic or aplastic transverse sinuses may mimic venous sinus thrombosis on MR venography or CT venography. Similarly, enlarged occipital sinuses or accessory venous channels may be misinterpreted as pathological findings. Awareness of these variants is therefore essential for accurate diagnosis and surgical planning.

### Limitations

This study has several limitations. First, the sample size was relatively small (*n* = 15), which is inherent to cadaveric anatomical studies and limits generalizability. Second, all specimens were obtained from elderly individuals, which may not reflect variations in younger populations. Third, formalin fixation may result in tissue shrinkage, potentially affecting morphometric measurements. Finally, no radiological correlation [e.g., magnetic resonance venography (MRV) or computed tomography venography (CTV)] or clinical history was available.

## Conclusion

The wide spectrum of anatomical variations identified in our study underscores the importance of careful preoperative imaging for procedures involving the posterior fossa, tentorium, or parasagittal regions. The presence of dural folds in the CS region, asymmetric TS dominance, non-standard StS insertions, and enlarged OS channels may significantly alter surgical landmarks and risk profiles. Awareness of accessory sinuses and enlarged OSs may prevent misinterpretation during MRV or CT venography.

Our findings support the view that the anatomy of the dural venous sinuses exhibits considerably greater variability than traditionally depicted. Rare variations in dural venous sinus anatomy can fundamentally alter surgical management, and recognizing such variations is crucial to minimizing the risk of potentially fatal complications. Dural venous sinus variants can frequently be mistaken for thrombosis, particularly in patients with neurological complaints, as venous thrombosis typically involves multiple sinuses. Consequently, detailed knowledge of the prevalence and interrelationships of these variations is essential to ensure accurate differentiation from pathological entities such as thrombosis and to minimize diagnostic error. Our results contribute to existing anatomical knowledge and underscore the clinical importance of accounting for such variability in both diagnostic and surgical contexts.

## Data Availability

The original contributions presented in the study are included in the article/Supplementary Material, further inquiries can be directed to the corresponding author. The corresponding author can be contacted for original contributions presented in this study.
